# Supplementary material for: Androgen-Induced MIG6 Regulates Phosphorylation of Retinoblastoma Protein and AKT to Counteract Non-Genomic AR Signaling in Prostate Cancer Cells
Source: Biomolecules. 2022 Jul 29;12(8):1048. doi: 10.3390/biom12081048 (PMC9405759; doi:10.3390/biom12081048)
Supplement: Supplementary file 1 [file biomolecules-12-01048-s001.zip › biomolecules-1586393-supplementary.pdf]

Figure S1. List of the 33 genes upregulated upon SAL overlapping with LNCaP and C4-2 cells.

STEAP4	*SAT1*
AKAP12	*SLC2A3*
MAF	*CTNNA2*
ERRFI1	*SOCS2*
CCDC141	*GNMT*
MYBPC1	*RHOU*
NDRG1	*USP50*
MOGAT2	*SMS*
TBX15	*TG*
HPGD	*SLC35F1*
SLC15A2	*AGR2*
ACSM1	*REP15*
POTEI	*PTPN21*
ZNF812	*LIFR*
PHLDB2	*ST6GALNAC1*
SNAI2	*TMCC3*
UGT2B28	

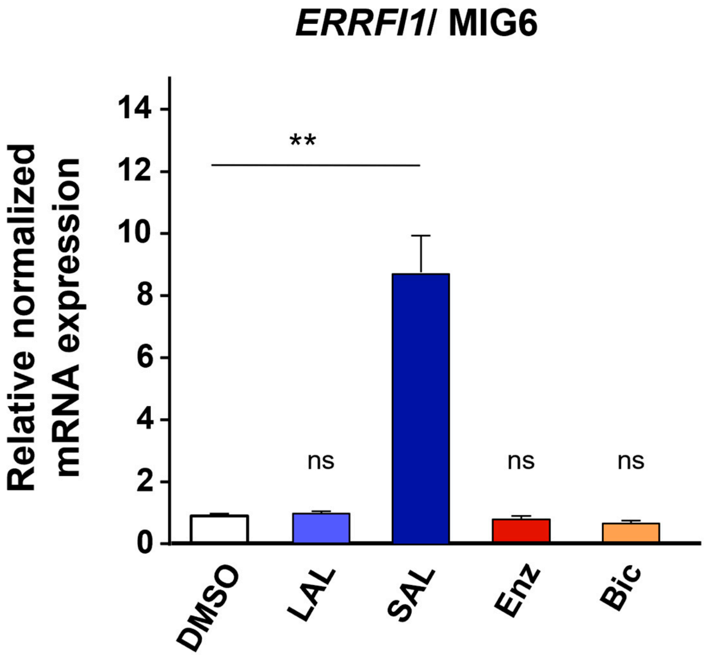

Figure S2. Treatment with AR antagonists do not induce *ERRFI1* expression. LNCaP were treated for 72 hours. The expression of *ERRFI1* was analyzed by qRT-PCR. Bicalutamide (BIC) and Enzalutamide (Enz) were used at 10μM final concentration. LAL, 1pM R1881; SAL, 1nM R1881.

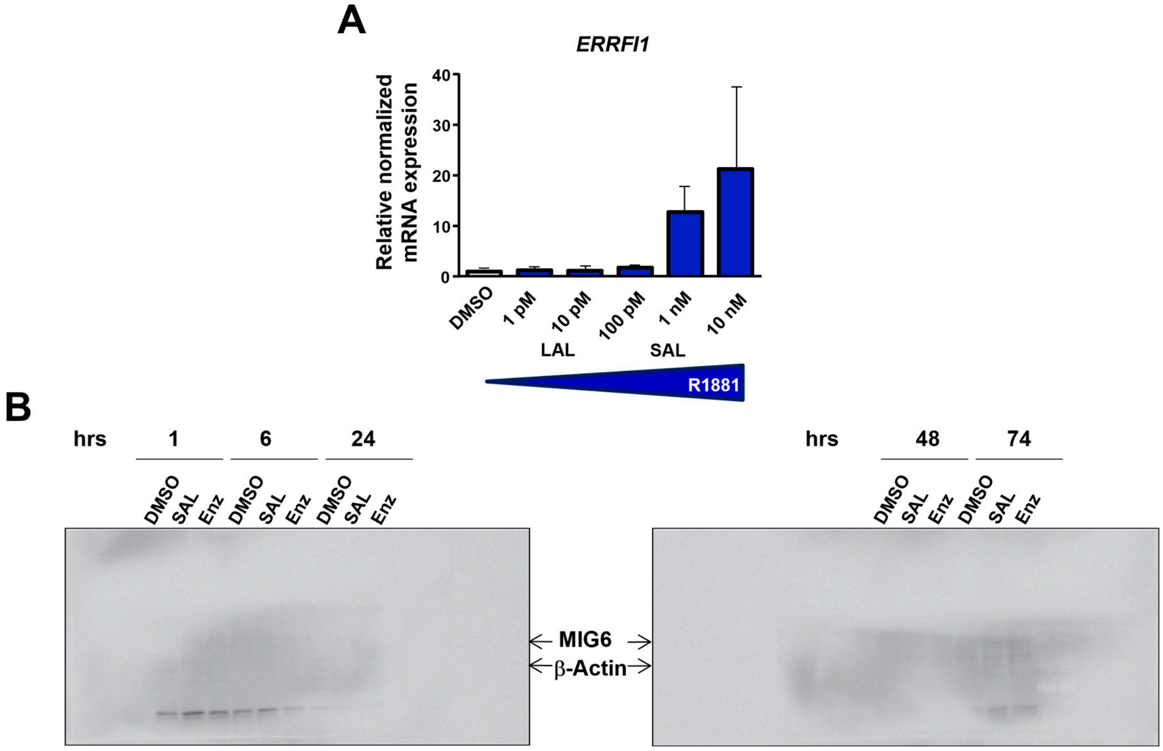

Figure S3. Expression change of *ERRFI1* by androgen dose and incubation time. A) Dose-dependent androgen treatment indicates expression of *ERRFI1* is induced at SAL levels. qRT-PCR were performed as shown in Fig. 2. Indicated are the R1881 concentrations and the correlation to LAL and SAL

based on Roediger et al. (2014). **B**) Induction of MIG6 protein levels by SAL and not by Enzalutamide (Enz) at various time points. LNCaP cells were treated with the indicated AR ligands. Protein extracts were performed at the indicated time points and analyzed by Westren blotting for MIG6 expression.

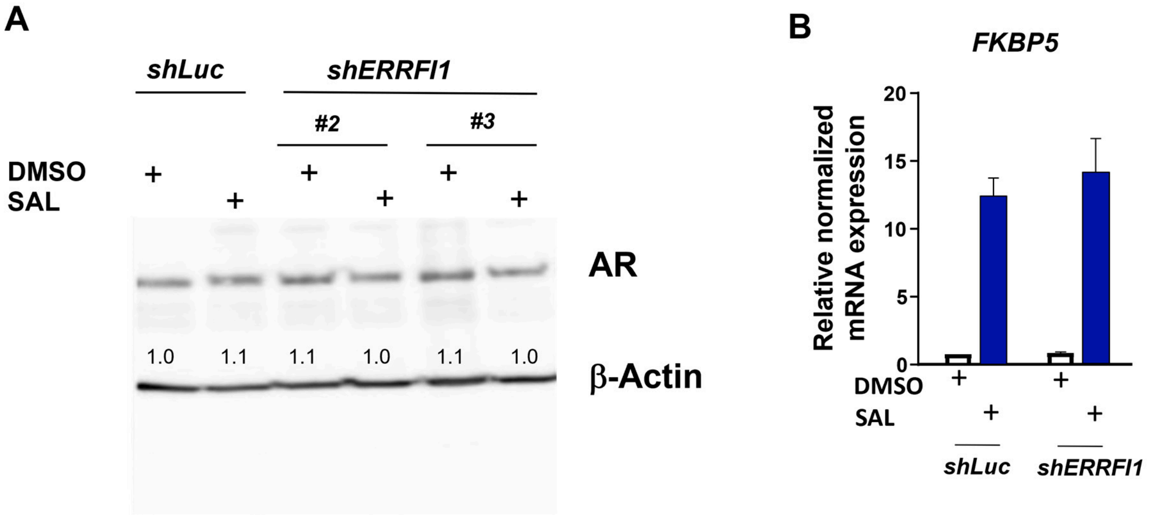

Figure S4. AR protein level and the AR target gene *FKBP5* are not affected by knockdown of *ERRFI1* / MIG6. Western Blot of LNCaP extracts transfected with shMig6#2, shMig6#3, or shLuc as control treated for 72 h with SAL or DMSO as solvent control. AR protein levels were detected by Western blot and the AR target gene *FKPB5* by qRT-PCR.

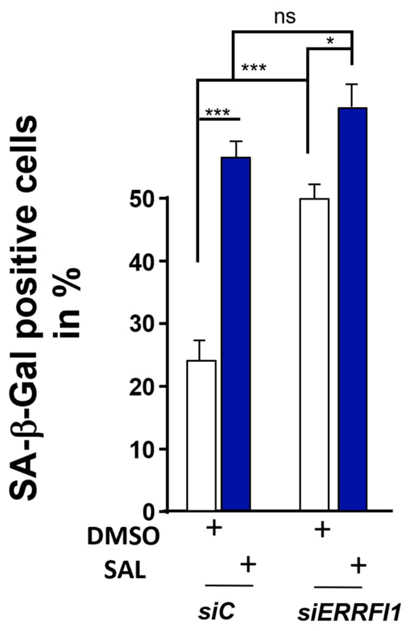

Figure S5. Similar to sh-mediated knockdown the si-mediated knockdown of *ERRFI1 induced cellular senescence.* SA-beta Gal staining of LNCaP transfected cells with si scrambled (siC) as control or si*ERRFI1* treated for 72 h with SAL or DMSO as solvent control.

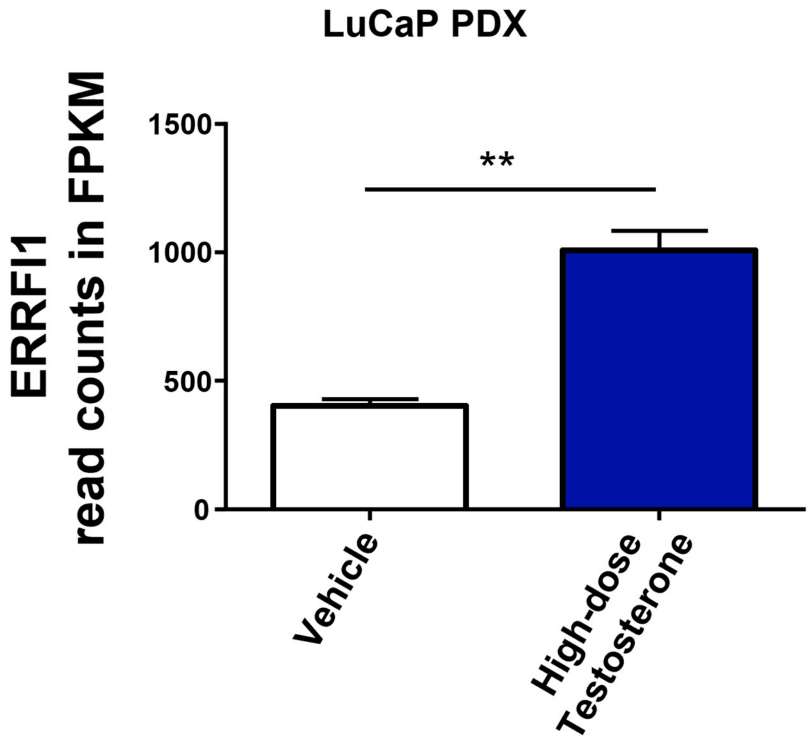

Figure S6. The expression of ERRFI1 is upregulated upon high-dose of testosterone treatment of mice in patient-derived xenografts using the LuCaP 35CR model system. Data were obtained from Han et al. (2022; GSE179687). The CRPC LuCaP patient-derived xenografts (PDX) model were analyzed by RNA-seq in castrated SCID mice treated with either vehicle or high-dose testosterone. FKPM: fragments per kilo base pair transcript per million reads.
